# A multilevel analysis of LGBT-relevant laws and sexual and gender minority mental health: evidence for the protective role of inclusive policies

**DOI:** 10.1186/s12889-026-26309-4

**Published:** 2026-01-30

**Authors:** Artur Luz Nunes Queiroz, Zhuo Meng, Casey D. Xavier Hall, Brittany Lane, Liying Wang, Rasheda Haughbrook, Carli Culjat, Amanda Gabster, Frank Wong, Eugenia Flores Millender, Umedjon Ibragimov

**Affiliations:** 1 Center of Population Sciences for Health Empowerment, Florida State University, 2010 Levy Avenue, Building B, Suite 3600, Tallahassee, FL 32310 USA; 2 College of Nursing, Florida State University, 98 Varsity Way, Tallahassee, FL 32306 USA; 3 Department of Psychology, Florida State University, 1107 W Call St, Tallahassee, FL 32306 USA

**Keywords:** Sexual and gender minorities, Mental health, Stress, Psychological, Health policy, Social determinants of health, Depression

## Abstract

**Background:**

Sexual and gender minority (SGM) individuals experience elevated rates of depression and anxiety, largely attributed to chronic exposure to stigma, discrimination, and social exclusion. Less is known about whether state-level policy climates may buffer the mental health impacts of stress among SGM populations.

**Methods:**

We used data from the All of Us Research Program (2020–2022) to examine whether state-level LGBTQ policy climates; measured using the Movement Advancement Project (MAP) Total Tally Score (range: –18.5 to 38.5), moderated associations between general perceived stress (PSS-10) and symptoms of depression and anxiety. Multilevel modeling assessed within- and between-state associations and cross-level interactions between policy climate and stress. Subgroup analyses tested whether moderation effects varied across demographic groups (e.g., marital status, gender identity). Final samples included *N* = 6,430 (depression) and *N* = 6,392 (anxiety).

**Results:**

A more inclusive state LGBTQ policy climate significantly moderated the association between perceived stress and mental health symptoms, reflecting a weaker stress–depression association (β = –.023, SE = .010, *p* = .020) and a weaker stress–anxiety association (β = –.032, SE = .009, *p* < .001). Moderation was stronger for divorced SGM individuals compared with married individuals (β = –.091, SE = .033, *p* = .006).

**Conclusion:**

State-level LGBTQ policy climates were statistically associated with variation in the strength of stress–mental health relationships among SGM adults. These findings highlight the relevance of structural environments for SGM mental health. Future longitudinal research should evaluate how changes in policy climates shape stress processes and mental health trajectories over time.

**Supplementary Information:**

The online version contains supplementary material available at 10.1186/s12889-026-26309-4.

## Background

Sexual and gender minority (SGM) individuals experience disproportionately higher rates of depression and anxiety compared to heterosexual and cisgender peers, with prevalence estimates 2–5 times greater [1–3]. These disparities are driven by unique stressors tied to stigma, discrimination, and lack of social support, which manifest in both overt prejudice and subtle microaggressions, ultimately harming mental health [4, 5].

To better understand the link between these stressors and mental health outcomes, the minority stress framework provides a valuable perspective. This framework posits that populations like SGM experience reduced social status which results in chronic exposure to prejudice, discrimination, and social rejection [6]. These exposures lead to cumulative stress, which contributes to poor mental health outcomes [6–8]. Minority stressors are conceptualized at multiple levels of the social ecology and applications of the framework since its inception have further articulated the role of the policy environment in either codifying differential social status (e.g. historical policies banning sex between consenting adults of the same sex) or buffering against differential social status (e.g., marriage equality or discrimination protections) [9]. Policies therefore have the potential to either exacerbate or disrupt minority stressors and their effect on cumulative stress and health.). Recognizing the mechanisms behind minority stress helps highlight why SGM individuals are disproportionately affected and informs strategies to mitigate its impact. In this study, we conceptualize policy not as the presence of discrete “protective” or “harmful” laws but as an overarching policy climate. The Movement Advancement Project (MAP) index summarizes both inclusive and restrictive laws, capturing the cumulative legal environment SGM individuals inhabit. This aligns with structural stigma scholarship showing that the aggregate policy climate (rather than any single statute) is a key driver of mental health [10].

Although minority stress theory distinguishes distal (structural/external) and proximal (identity-specific, intrapsychic) stressors, the present study focuses on the structural (distal) level of this framework (Meyer, 2003). Distal factors such as laws and institutional practices are theorized to shape mental health predominantly through proximal psychological processes (e.g., expectations of rejection, identity concealment, internalized stigma) that develop over time [8, 11]. Because measures of these proximal minority stress processes were not available in the All of Us dataset, we test whether the state-level policy climate moderates the association between general perceived stress and internalizing symptoms ie., whether living in a more inclusive policy climate buffers the impact of stress on depression and anxiety. We emphasize that this isolates the structural component of minority stress; proximal processes are likely mediators linking policy climates to individual mental health [8, 11].

Recent scholarship situates this within a broader “social safety” perspective [12], emphasizing that policies are not only symbolic markers of inclusion or exclusion but also material determinants of access to healthcare, legal protections, and supportive environments. Policies therefore shape whether SGM individuals can meet basic needs, maintain social connections, and access affirming care; conditions that directly influence stress and mental health outcomes.

Nonetheless, this body of work is often conducted without systematically addressing how and in what way broader macro-level or structural factors such as federal and/or state policies may influence or contribute to minority stress and its sequelae. The U.S. federalist system creates a patchwork of policies their federal laws provide overarching protection, state governments retain substantial autonomy to legislate healthcare, education [13], and civil rights matters. As a result, policies affecting SGM individuals can vary significantly from state to state, influencing their legal protections, access to healthcare, and overall quality of life. For example, some states enact policies that could magnify minority stress by exacerbating disparities in legal protections and healthcare access for SGM individuals, while others enact inclusive laws (e.g., anti-discrimination protections, gender-affirming care access) [5]. These policy differences translate to uneven lived experiences: protective policies correlate with lower psychological distress, while restrictive policies amplify minority stress [14, 15]. The dynamic nature of LGBTQ-related policy shifts further complicates this landscape. Policy changes between 2020 and 2022; including restrictions on gender-affirming care, education policies targeting sexual and gender diversity, and changes in insurance coverage, mean that the legal climate experienced by participants may differ from the MAP score used at baseline. This temporal mismatch is a recognized limitation in structural stigma research [10].

Despite this evidence, gaps remain. Much existing research has focused on proximal stressors (e.g., internalized stigma, concealment) or on broad associations between policy climate and mental health [14, 16]. Few studies have directly tested whether state-level policy environments moderate the fundamental relationship between perceived stress and mental health outcomes [10]. Moreover, little is known about whether these effects vary across demographic subgroups, such as marital status or gender identity. To address these gaps, the current study uses data from the All of Us Research Program (2020–2022) to examine whether state-level LGBTQ + policy inclusivity moderates the association between perceived stress and depression/anxiety among SGM individuals. Although psychological outcomes often show low between-state intraclass correlations, recent multilevel methodological work demonstrates that meaningful cross-level moderation can exist even when intercept variance is small [17, 18]. This is because contextual factors may influence how strongly stress translates into symptoms rather than average symptom levels themselves. Thus, MLM is necessary to test our core hypothesis regarding conditional associations across policy contexts. We hypothesize that higher policy inclusivity will buffer the impact of stress on mental health symptoms and that this buffering effect may vary across demographic subgroups such as marital status and gender identity.

In this study, we examine the moderating effect of state-level LGBTQ-relevant laws on the association between general perceived stress and mental health outcomes. These processes likely mediate how policy environments shape mental health but were unavailable in All of Us. Accordingly, the present study evaluates structural buffering rather than the full minority stress pathway.

## Methods

### Participants

The study was conducted using data from the All of Us Research Program (AoU) Controlled Tier Dataset v7 (May 2017 to July 2022) (All of Us Research Program Investigators, 2019) [19]. Given the state equity policy data, one of the key predictors of interest was collected and calculated as of January 1, 2020. sexual and gender minority (SGM) participants who enrolled after 2020 with their depression, anxiety, stress, and zip code data available were eligible for the current study. U.S. territories were excluded due to unavailability of state-level policy data. The last mental health observation of the year was selected when multiple observations occurred within a year. For participants with data available at more than one point, baseline data was used for the cross-sectional analysis. The final sample for depression consisted of 6,430 participants from 2020 to 2022. The sample for anxiety consisted of 6,392 participants from 2020 to 2021.

Secondary analyses utilizing the Controlled Tier were determined not to constitute research involving human subjects (2021–02-TN-001).

Because state policy data were available as of January 1, 2020, we included SGM participants who enrolled after 2020 and had available data on depression, anxiety, stress, and zip code. U.S. territories were excluded due to unavailable policy data. For individuals with multiple assessments in a single year, the last observation was used; when multiple years were available, baseline measures were used. The analytic sample included 6,430 participants for depression and 6,392 for anxiety.

### Measures

#### Outcomes

##### Depression

Depression symptoms were measured by the 9-item Patient Health Questionnaire (PHQ-9) and its abbreviated two-item version (PHQ-2). Data were extracted from the COVID-19 Participant Experience (COPE) survey and Electronic Health Record (EHR) domains within the All of Us (AoU) platform [20]. Participants rated the frequency of depressive symptoms over the past two weeks on a four-point Likert-type scale (0 = “Not at all” to 3 = “Nearly every day”). Total scores range from 0 to 27 for the PHQ-9 and 0 to 6 for the PHQ-2, with higher scores indicating greater symptom severity.

To harmonize the PHQ-9 and PHQ-2 scores into a single, continuous measure of depression symptom severity for analysis, total scores were converted to z-scores within each respective scale. This statistical standardization process (mean = 0, SD = 1) allows for the combined use of both measures in our models while maximizing statistical power by preserving the full continuous nature of the data.

##### Anxiety

Anxiety symptoms were assessed using the 7-item Generalized Anxiety Disorder scale (GAD-7) and its abbreviated two-item version (GAD-2), sourced from the COPE survey and HER [20, 21]. Items were rated on an identical four-point scale (0 = “Not at all” to 3 = “Nearly every day”). Total scores range from 0 to 21 for the GAD-7 and 0 to 6 for the GAD-2, with higher scores indicating greater symptom severity.

Following the same procedure as for depression, total scores were standardized within each scale (GAD-7 and GAD-2) to create a unified continuous measure of anxiety symptom severity for analysis.

### Key predictor

#### Perceived stress

We measured general perceived stress using the 10-item Perceived Stress Scale (PSS-10), which assesses respondents’ appraisal of life as unpredictable, uncontrollable, and overloaded over the past month [22]. The PSS-10 is a global indicator of perceived stress and is not a measure specific to minority stress processes (e.g., internalized stigma, concealment). The PSS-10 is a global indicator of perceived stress and is not a measure specific to minority stress processes (e.g., internalized stigma, concealment). Therefore, while elevated PSS-10 scores among SGM participants may reflect the burden of minority stressors, the PSS-10 does not isolate identity-specific stress and should be interpreted as a general stress measure.

#### Demographics

Age, gender identity (GI), sexual orientation (SO), race/ethnicity, nativity, education, annual household income, health insurance, marital status, and state from AoU were included in the study. Each demographic variable’s categories can be found in Table 1, and their detailed descriptions are available in Supplemental Table S1.

**Table 1:** Descriptive statistics of individual- and state-level covariates for depression and anxiety among All of Us participants from 2020 to 2022

Individual level covariates	Depression ($$N$$= 6,430)	Anxiety ($$N$$= 6,392)	
Stress	16.356 (8.226)	16.355 (8.230)	
Age			
No more than 40 years	2634 (41.0%)	2612 (40.9%)	
40–65 years	2645 (41.1%)	2638 (41.3%)	
Over 65 years	1151 (17.9%)	1142 (17.9%)	
Gender Identity			
Cis-gender man	2359 (36.7%)	2350 (36.8%)	
Cis-gender woman	3361 (52.3%)	3340 (52.3%)	
Non-binary	410 (6.4%)	405 (6.3%)	
Transgender	257 (4.0%)	254 (4.0%)	
Other	43 (.7%)	43 (.7%)	
Sexual Orientation			
Heterosexual	107 (1.7%)	105 (1.6%)	
Homosexual	2836 (44.1%)	2825 (44.2%)	
Bi-sexual	2824 (43.9%)	2807 (43.9%)	
Asexual	181 (2.8%)	178 (2.8%)	
Queer	216 (3.4%)	214 (3.3%)	
Other	235 (3.7%)	233 (3.6%)	
Multi	31 (.5%)	30 (.5%)	
Race/ethnicity			
Non-Hispanic White	5093 (79.2%)	5065 (79.2%)	
Non-Hispanic Black	304 (4.7%)	305 (4.8%)	
Non-Hispanic Asian&NHPI	219 (3.4%)	217 (3.4%)	
Non-Hispanic another race	88 (1.4%)	88 (1.4%)	
Hispanic	538 (8.4%)	531 (8.3%)	
Multi race/ethnicity	188 (2.9%)	186 (2.9%)	
Nativity			
U.S. born	5898 (91.7%)	5863 (91.7%)	
Foreign born	532 (8.3%)	529 (8.3%)	
Education			
Less than a college degree	1958 (30.5%)	1944 (30.4%)	
College graduate	2148 (33.4%)	2132 (33.4%)	
Advanced degree	2324 (36.1%)	2316 (36.2%)	
Income			
Less than $35,000	1757 (27.3%)	1749 (27.4%)	
$35,000 – $74,999	1699 (26.4%)	1690 (26.4%)	
$75,000 – $149,999	1687 (26.2%)	1674 (26.2%)	
$150,000 and more	989 (15.4%)	983 (15.4%)	
Missing/prefer not to answer	298 (4.6%)	296 (4.6%)	
Health Insurance			
Private	3101 (48.2%)	3081 (48.2%)	
Public	1445 (22.5%)	1437 (22.5%)	
Both	528 (8.2%)	522 (8.2%)	
Other/none	168 (2.6%)	168 (2.6%)	
Missing/prefer not to answer	1188 (18.5%)	1184 (18.5%)	
Marital Status			
Married	2115 (32.9%)	2101 (32.9%)	
Live with partner	991 (15.4%)	982 (15.4%)	
Separated	104 (1.6%)	104 (1.6%)	
Divorced	608 (9.5%)	601 (9.4%)	
Widowed	142 (2.2%)	140 (2.2%)	
Never married	2470 (38.4%)	2464 (38.5%)	
State level covariates	Summary 2020	Summary 2021	Summary 2022
State policy (range: −18.5 to 38.5)	13.843 (14.220)		
Mental health facility ratio	56.390 (27.082)	50.188 (24.115)	45.764 (22.488)
Total population in million	6.499 (7.408)	6.465 (7.371)	6.492 (7.392)
Median age	38.559 (2.394)	38.641 (2.323)	38.810 (2.287)
Sex ratio	97.665 (3.299)	98.643 (3.267)	99.025 (3.387)
Partisan control			
Democracy	16 (31.4%)	16 (31.4%)	15 (29.4%)
Republic	22 (43.1%)	24 (47.1%)	24 (47.1%)
Divided	13 (25.5%)	11 (21.6%)	12 (23.5%)

The summary statistics are Mean (SD) or Count (%). Total percentage not equaling 100% is due to rounding

#### State-level covariates

##### State policy

We used the Movement Advancement Project (MAP) Total Tally Score (2020) as our state-level indicator of the LGBTQ policy environment. The MAP score is a composite tally that aggregates the presence of multiple laws and policies across domains (e.g., nondiscrimination, healthcare access, parental rights), assigning positive values to inclusive policies and negative values to restrictive policies (Movement Advancement Project, 2020). As of January 1, 2020, nearly 40 LGBTQ-related laws and policies were tracked by MAP within the 50 U.S. states and Washington, D.C., and the five U.S. territories. A score (somewhere between −1 and 1) was assigned to each policy based on its level of inclusiveness or exclusiveness, with higher scores indicating greater inclusivity and more legal protections for LGBTQ + individuals or lower structural stigma, leading to a total tally score ranging from −18.5 to 38.5 (see Supplemental Table S3). A higher tally indicated a more LGBTQ-inclusive and protective environment (e.g., more anti-discrimination laws, gender-affirming care access). The MAP index captures a composite LGBTQ policy climate based on both protective and restrictive state laws. Because the index aggregates policies of varying directionality, it reflects the overall inclusivity or restrictiveness of the policy environment but cannot disentangle the independent effects of pro- versus anti-LGBT policies.

##### Demographics and confounders

State-level population size, median age, sex ratio (males per 100 females), partisan control, and mental health facility ratio (facilities per 1 million residents) were included to adjust for potential confounding at the state level. Their definitions, categories, and data sources are provided in Table 1 and Supplemental Table S1.

### Statistical analysis

Individual- and state-level data were merged by state. Descriptive statistics and bivariate regression analysis were conducted to capture the sample characteristics. Multilevel modeling with a restricted maximum likelihood (REML) estimator was used to assess the effect of LGBTQ-related laws and policies on mental health outcomes. The unconditional model (Model 0) was first applied to evaluate the random effects. Next, three models were gradually tested: Model 1 contained only individual-level covariates; Model 2 contained both individual- and state-level covariates; Model 3 further contained the cross-level interaction between state policy and stress to evaluate the moderation effect of state policy on the relationship between stress and mental health. The interactive relationship was additionally investigated in subgroups. All continuous covariates were first standardized. Cluster mean centering and grand mean centering were adopted at the individual and group levels, respectively. An aggregate of the cluster mean-centered variable was introduced to the analysis. The reference categories for all categorical variables are indicated in the result tables. Participants who preferred not to answer or did not report their income or health insurance were categorized as “Missing” and retained in the analysis. Those with missing data on other individual-level demographics were excluded (about 3.3%). All analyses were conducted using R within the AoU workbench. Multilevel models were estimated using the “lme4” package [23]. We report outcome ICCs and Level-1 slope variance (T11) in Table 2 and assessed sensitivity to observed slope variability following Mathieu et al. (2012). Although between-state variance was small, this pattern is typical for psychological outcomes and underscores that policy context functions as a modifier of stress-related risk rather than a primary source of outcome variance. Our primary hypothesis concerned whether the stress–mental health slope, rather than the intercept, varied by policy climate. Multilevel modeling is therefore appropriate despite minimal between-state variance.

**Table 2:** Results of multilevel modeling predicting depression and anxiety among All of Us participants from 2020 to 2022

	Depression ($$N$$= 6,430)				Anxiety ($$N$$= 6,392)			
	Model 0	Model 1	Model 2	Model 3	Model 0	Model 1	Model 2	Model 3
Covariates		$$\beta$$(SE)	$$\beta$$(SE)	$$\beta$$(SE)		$$\beta$$(SE)	$$\beta$$(SE)	$$\beta$$(SE)
Stress		.599*** (.011)	.601*** (.011)	.610*** (.011)		.626*** (.010)	.628*** (.010)	.641*** (.010)
State average stress			.730*** (.109)	.730*** (.109)			.699*** (.109)	.699*** (.109)
State policy			-.012 (.019)	-.013 (.019)			-.009 (.021)	-.010 (.021)
Stress$$\times$$State policy				-.023* (.010)				-.032*** (.009)
Age								
No more than 40 years		ref	ref	ref		ref	ref	ref
40–65 years		-.001 (.024)	-.007 (.024)	-.006 (.024)		-.120*** (.023)	-.117*** (.023)	-.116*** (.023)
Over 65 years		-.094** (.036)	-.090* (.036)	-.089* (.036)		-.199*** (.035)	-.196*** (.035)	-.195*** (.035)
Gender identity								
Cis-gender man		ref	ref	ref		ref	ref	ref
Cis-gender woman		-.040 (.023)	-.044 (.023)	-.044 (.023)		.060** (.022)	.056* (.022)	.056* (.022)
Non-binary		.123** (.045)	.124** (.045)	.126** (.045)		.126** (.044)	.123** (.044)	.127** (.044)
Transgender		.089 (.055)	.077 (.055)	.079 (.055)		.104 (.054)	.093 (.054)	.094 (.054)
Other		.158 (.118)	.149 (.118)	.146 (.118)		.073 (.114)	.062 (.114)	.058 (.114)
Sexual orientation								
Heterosexual		ref	ref	ref		ref	ref	ref
Gay/Lesbian		.142 (.083)	.133 (.083)	.133 (.083)		.057 (.081)	.049 (.081)	.048 (.081)
Bi-sexual		.161 (.082)	.149 (.082)	.150 (.082)		.032 (.081)	.021 (.080)	.022 (.080)
Asexual		.160 (.098)	.144 (.097)	.145 (.097)		-.034 (.095)	-.050 (.095)	-.048 (.095)
Queer		.177 (.093)	.166 (.093)	.168 (.093)		.030 (.091)	.020 (.091)	.023 (.090)
Other		.158 (.093)	.148 (.093)	.149 (.093)		.057 (.091)	.047 (.091)	.048 (.091)
Multi		-.027 (.157)	-.033 (.156)	-.041 (.156)		-.118 (.154)	-.124 (.154)	-.137 (.154)
Race/ethnicity								
Non-Hispanic White		ref	ref	ref		ref	ref	ref
Non-Hispanic Black		-.030 (.046)	-.022 (.046)	-.022 (.046)		-.066 (.044)	-.053 (.044)	-.052 (.044)
Non-Hispanic Asian&NHPI		-.094 (.056)	-.090 (.055)	-.089 (.055)		-.089 (.054)	-.082 (.054)	-.081 (.054)
Non-Hispanic another race		-.041 (.081)	-.043 (.081)	-.037 (.081)		-.019 (.079)	-.017 (.079)	-.008 (.079)
Hispanic		-.073* (.037)	-.067 (.036)	-.064 (.036)		-.057 (.036)	-.052 (.035)	-.048 (.035)
Multi race/ethnicity		.003 (.056)	.035 (.056)	.036 (.056)		-.081 (.055)	-.077 (.055)	-.076 (.055)
Nativity								
U.S. born		ref	ref	ref		ref	ref	ref
Foreign born		-.077* (.038)	-.075* (.038)	-.076* (.038)		-.064 (.037)	-.060 (.036)	-.061 (.036)
Education								
Less than a college degree		ref	ref	ref		ref	ref	ref
College graduate		-.088*** (.025)	-.083*** (.025)	-.082*** (.025)		-.045 (.024)	-.040 (.024)	-.038 (.024)
Advanced degree		-.071** (.026)	-.066* (.026)	-.066* (.026)		-.040 (.025)	-.035 (.025)	-.035 (.025)
Annual household income								
Less than $35,000		ref	ref	ref		ref	ref	ref
$35,000 – $74,999		-.022 (.028)	-.018 (.028)	-.017 (.028)		-.007 (.027)	-.002 (.027)	-.001 (.027)
$75,000 – $149,999		-.059 (.031)	-.051 (.031)	-.049 (.031)		-.050 (.030)	-.041 (.030)	-.040 (.030)
$150,000 and more		-.062 (.037)	-.045 (.037)	-.046 (.037)		-.055 (.036)	-.039 (.036)	-.040 (.036)
Missing/prefer not to answer		-.054 (.048)	-.041 (.048)	-.038 (.048)		-.050 (.046)	-.037 (.046)	-.032 (.046)
Health insurance								
Private		ref	ref	ref		ref	ref	ref
Public		.091** (.029)	.091** (.029)	.091** (.029)		.034 (.028)	.034 (.028)	.034 (.028)
Both		.143*** (.040)	.143*** (.040)	.142*** (.040)		.045 (.039)	.044 (.039)	.043 (.039)
Other/none		-.010 (.061)	-.006 (.060)	-.007 (.060)		-.059 (.059)	-.055 (.059)	-.056 (.059)
Missing/prefer not to answer		.015 (.027)	.012 (.027)	.012 (.027)		-.008 (.026)	-.012 (.026)	-.011 (.026)
Marital status								
Married		ref	ref	ref		ref	ref	ref
Live with partner		.023 (.030)	.027 (.030)	.029 (.030)		-.017 (.029)	-.013 (.029)	-.010 (.029)
Separated		.055 (.076)	.062 (.076)	.061 (.076)		-.047 (.074)	-.045 (.074)	-.047 (.074)
Divorced		.143*** (.036)	.145*** (.036)	.144*** (.036)		-.028 (.035)	-.023 (.035)	-.024 (.035)
Widowed		.181** (.066)	.189** (.066)	.189** (.066)		.019 (.065)	.027 (.065)	.027 (.064)
Never married		.088*** (.025)	.094*** (.025)	.094*** (.025)		-.057* (.025)	-.051* (.025)	-.053* (.025)
State mental health facility ratio			.016 (.022)	.016 (.022)			.034 (.023)	.034 (.023)
State total population			.004 (.010)	.004 (.010)			.011 (.011)	.011 (.011)
State median age			.006 (.017)	.006 (.017)			-.007 (.018)	-.007 (.018)
State sex ratio			.027 (.020)	.027 (.020)			.016 (.021)	.016 (.021)
State partisan control								
Democratic			ref	ref			ref	ref
Republican			-.007 (.048)	-.007 (.048)			-.018 (.052)	-.018 (.052)
Divided			-.028 (.037)	-.028 (.037)			-.045 (.040)	-.044 (.040)
$${R}^{2}$$Marginal	.000	.410	.419	.420	.000	.453	.459	.460
$${R}^{2}$$Conditional	.005	.417	.419	.420	.004	.457	.460	.461
AIC	17970	14599	14548	14547	17919	14101	14059	14052
BIC	17990	14849	14853	14865	17939	14351	14364	14369
ICC	.005	.011	.000	.000	.004	.008	.001	.001

*AIC* Akaike Information Criterion, *BIC* Bayesian Information Criterion, *ICC* Intraclass Correlation Coefficient

^*^p<.05, **p<.01, ***p<.001

## Results

### Sample characteristics

Overall, participants were at a moderate level of stress (between 14 and 26). At the individual level, about 41% of the participants were in early and midlife, while the rest 18% were in their late adulthood. Most of the participants were cis-gender men (36.7%) and women (52.3%), and self-identified as gay/lesbian (44.1%) or bi + (43.9%). Regarding race/ethnicity and nativity, most SGM were non-Hispanic white (79.2%) and U.S. born (91.7%). The participants’ education level and annual household income were evenly distributed across the corresponding categories. For health insurance, 48.2% and 22.5% of participants had private and public insurance, respectively. Sample characteristics of these SGM participants are shown in Table 1.

The rest 8.2% had both, while 2.6% had other plans or no insurance. For marital status, over a third of SGM participants were never married; about 32.9% were married and 15.4% were living with their partners. The associations between these individual-level covariates and depression and anxiety are summarized in Supplemental Table S2. At the state level, the average state policy tally score was 13.8 (SD = 14.2), indicating a level of ‘fair’ (Movement Advancement Project, 2020). The number of eligible mental health facilities per 1 million people averaged 56.390 in 2020, which decreased in 2021 and 2022. On average, the total population was about 6.5 million (SD = 7.4), and the median age was about 39 years (SD = 2.3) throughout these three years. The average number of males per 100 females was about 98 in 2020 (SD = 3.3) and increased in the following two years. As for state control, there were more Republican states than Democratic states; about a quarter of the states had divided control.

### Multilevel modeling results

Table 2 presents the multilevel modeling results. Participants per state ranged from 3 to 1,113 (see Supplemental Table S3). The intraclass correlation coefficient (ICC) from the null model (Model 0) was 0.005 for depression and 0.004 for anxiety, indicating that only a limited proportion of the total variance in mental health outcomes was attributable to differences between states. Substantively, this suggests that state-level policy context is a relatively modest driver of overall variation in depression and anxiety compared with individual-level factors or more proximal environments (e.g., interpersonal, community, or healthcare contexts). This pattern is common in multilevel research on psychological outcomes, where most variance resides at the individual level rather than the contextual level. Importantly, low between-state variance does not preclude meaningful contextual moderation: state policy climate may still shape how individual stress translates into mental health symptoms by altering the conditions under which stress is experienced. Accordingly, our findings should be interpreted as evidence that policy environments function as contextual modifiers of stress-related risk, rather than as primary determinants of mental health status. Future research incorporating more proximal geographic units (e.g., counties or municipalities) and longitudinal designs may further clarify how structural contexts at multiple levels contribute to mental health disparities among sexual and gender minority populations [17, 18]. Despite the small ICCs, we retained multilevel modeling to account for the nested data structure and to test the hypothesized cross-level interactions [24]. The model with individual-level covariates only (Model 1) showed that after adjusting for demographics, stress was positively associated with both depression (β = 0.599, SE = 0.011, *p* < 0.001) and anxiety (β = 0.626, SE = 0.010, *p* < 0.001). About 41% and 45% of the variances in depression and anxiety were explained by the model, respectively. Adding state-level covariates to the model (Model 2) increased *R*^2^ and reduced Akaike Information Criterion (AIC), suggesting improved model fit. We prioritized the AIC when comparing models, as it is less punitive of model complexity than the BIC (particularly in large samples) and aligns with the theory-driven purpose of evaluating the cross-level interaction. The positive effect of within-state stress on depression (β = 0.601, SE = 0.011, *p* < 0.001) and anxiety (β = 0.628, SE = 0.010, *p* < 0.001) remained significant, as did the state-average stress on depression (β = 0.730, SE = 0.109, *p* < 0.001) and anxiety (β = 0.699, SE = 0.109, *p* < 0.001). In contrast, state policy was not significantly associated with either outcome. Model 3 which incorporated the cross-level interaction between stress and state policy increased both marginal and conditional *R*^2^ and reduced the AIC, indicating improved fit. However, the Bayesian Information Criterion (BIC) increased through models 1 to 3 for both depression and anxiety. Given our hypotheses and the goal of identifying the most predictive model, we adopted Model 3, which had the lowest AIC and accounted for more variance.

**Fig. 1:**
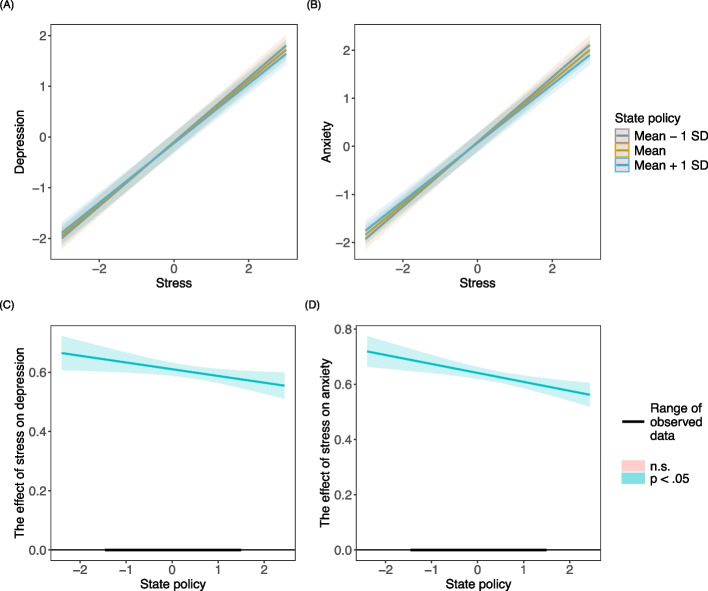
The moderating effect of state policy on the relationship between stress and mental health. *Note*. Panels (**A**) and (**B**) present the associations between stress and mental health by state policy. Panels (**C**) and (**D**) are the corresponding Johnson-Newman plots. The solid lines and shading represent the estimates and their 95% confidence intervals

Model 3 revealed distinct stress and mental health associations at different ecological levels. Holding all other covariates fixed, a one standard deviation (SD) increase in within-state stress was associated with a 0.610 (SE = 0.011, *p* < 0.001) SD increase in depressive symptoms, while a one SD increase in state-average stress was associated with a 0.730 (SE = 0.109, *p* < 0.001) increase in depressive symptoms. This pattern indicates that both individual stress experiences and broader state-level stress environments were each statistically associated with depressive symptoms among SGM individuals. The significant cross-level interaction (β = −0.023, SE = 0.010, *p* = 0.020) suggested that for two participants with the same stress level, the participant from a state with more inclusive LGBTQ + equity policies (one SD increase relative to the grand mean) would be linked to a 0.023 SD decrease in depressive symptoms. Figure 1(A) visualized the moderating effect. The Johnson–Newman plot (see Fig. 1(C)) was also created to directly illustrate that better state policies weakened the influence of stress on depressive symptoms.

For anxiety, a one SD increase in within-state stress and in state-average stress was associated with 0.641 (SE = 0.010, *p* < 0.001) and 0.699 (SE = 0.109, *p* < 0.001) increases in anxiety, respectively, when adjusting for other covariates. The coefficient of the interaction term was −0.032 (SE = 0.009, *p* < 0.001), indicating that the association between stress and anxiety symptoms was statistically weaker in states with more inclusive LGBTQ-related policy climates. The moderating effect was visualized in Fig. 1(B) and (D).

### Subgroup analysis results

**Fig. 2:**
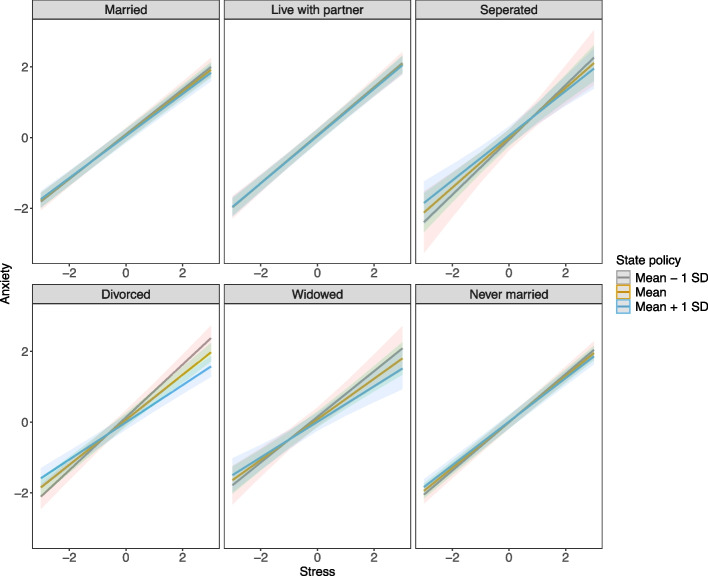
The moderating effect of state policy on anxiety in marital status subgroups. *Note.* The solid lines and shading represent the estimates and their 95% confidence intervals

We systematically analyzed whether the moderating effect differed across individual demographic groups by considering the three-way interactions in the model. The difference was only observed in marital status for the moderating effect of state policy on the relationship between stress and anxiety. Figure 2 showcased these moderating effects in marital status subgroups. Notably, the association between stress and anxiety symptoms was weaker in states with more inclusive policy climates (3-way interaction term: β = −0.091, SE = 0.033, *p* = 0.006). This particularly pronounced buffering effect may reflect how policy protections interact with social position to shape the intensity of minority stress; a dynamic consistent with Brooks’ emphasis on structural access and Diamond & Alley’s framing of policies as mechanisms that create or dismantle social safety. In summary, multilevel models indicated that inclusive state policies significantly buffered the negative association between stress and both depression and anxiety symptoms among SGM individuals. This moderation effect was particularly pronounced among divorced individuals, underscoring the importance of policy environments in shaping mental health disparities. These findings highlight the multilevel nature of minority stress and demonstrate that variability in state policy climates is statistically associated with differences in stress–mental health associations among SGM individuals.

## Discussion

This study contributes to the growing literature on minority stress and mental health by demonstrating that inclusive state-level policies can buffer the negative effects of perceived stress on depression and anxiety among sexual and gender minority (SGM) individuals. Using data from the All of Us Research Program (2020–2022), we found that more inclusive LGBTQ + policies significantly attenuated the association between stress and both depression and anxiety symptoms. These findings remained robust after adjusting for individual- and state-level covariates. In summary, our findings suggest that both individual-level and contextual stressors contribute independently to depression and anxiety symptoms among SGM individuals. Notably, inclusive state-level LGBTQ + policies were found to moderate these relationships, attenuating the psychological impact of perceived stress. This buffering effect was particularly strong among divorced SGM individuals, indicating that policy environments may offer more pronounced protection for certain socially vulnerable subgroups. These results underscore the importance of addressing both structural and psychosocial determinants of mental health in interventions and policy design aimed at reducing disparities within SGM populations.

This study extends minority stress theory by empirically testing how structural contexts (specifically, inclusive state-level LGBTQ + policies) moderate the relationship between perceived stress and mental health outcomes. Minority stress theory posits that SGM individuals are exposed to chronic, identity-related stressors rooted in stigma, including proximal stressors (e.g., internalized stigma, concealment, expectations of rejection) and distal stressors (e.g., interpersonal discrimination, legal inequality) [4, 5, 8, 25]. While most research has focused on proximal or interpersonal factors, fewer studies have assessed whether structural stigma, reflected in policy environments, amplifies or attenuates the mental health consequences of minority stress.

Minority Stress Theory [8] and subsequent empirical work on structural stigma [11] propose that distal, structural exposures (for example, discriminatory laws or an unsupportive policy climate) influence mental health primarily through proximal, identity-specific psychological processes (e.g., internalized stigma, vigilance, concealment). In other words, living under a hostile legal regime can increase expectations of rejection and identity concealment, which then escalate distress and depressive/anxious symptoms. Because the All of Us dataset did not include validated measures of these proximal constructs for the present sample, our analysis isolates the structural level: we test whether the broader policy climate moderates the general stress–mental health association. Accordingly, the observed buffering by an inclusive policy climate is most plausibly understood as evidence that structural environments shape the extent to which stress translates into psychopathology, with proximal minority stress processes likely mediating this effect. Future longitudinal work that includes both distal and proximal measures is needed to empirically test this mediation pathway [8, 11]. Because these proximal processes were unmeasured, our findings should be interpreted as identifying structural conditions under which general stress becomes more or less consequential for mental health, rather than estimating the full minority stress pathway. This analytic choice isolates the structural component of minority stress but cannot capture the psychological mechanisms through which policies exert their effects.

Our analysis of revealed three key findings. First, inclusive policies significantly attenuated the associations between perceived stress and both depression and anxiety symptoms among SGM individuals. These findings suggest that protective policy environments may weaken the internalization of stigma and reduce vigilance around potential rejection, thereby softening the impact of everyday stressors on psychological outcomes. This aligns with emerging work suggesting that inclusive policies are not only markers of social climate but also mechanisms that reshape the experience of minority stress itself [11, 26–29]. This finding complements recent theoretical developments that situate minority stress within a broader “social safety” framework [12]. This perspective emphasizes that policies are not merely symbolic markers of inclusion or exclusion; they also alter the material and social conditions that determine whether individuals can meet basic needs, maintain supportive networks, and access affirming care.

Second, these buffering effects remained robust after adjusting for individual-level demographics and state-level covariates. Notably, the relatively small between-state variance observed in our models suggests that state policy context is not a dominant driver of overall mental health variation, but rather operates as a higher-level condition that modifies how individual stress exposures translate into psychological symptoms. This finding suggests that the protective association exists independently of other, non-policy related factors, including residing in a politically “blue” or “red” state. Rather, it reflects the independent influence of the policy environment itself, which can vary considerably within states of similar partisan orientation [30]. This suggests that inclusive policies yield measurable mental health benefits beyond the broader political climate, underscoring the importance of examining specific legislative protections rather than relying solely on partisan control as a proxy for social climate [31]. Because the MAP index combines protective and restrictive laws, the observed moderation likely reflects variation in overall policy climate rather than the effect of protective policies alone. Future research should separately evaluate pro- and anti-LGBT policies to clarify mechanisms. Importantly, because the MAP index is an aggregate measure that incorporates both protective and restrictive statutes, our findings should be interpreted as reflecting the *overall policy climate* rather than the independent impact of affirming or discriminatory laws. This distinction is essential: structural stigma research emphasizes that it is the cumulative legal and symbolic environment (not any single policy) that shapes minority stress processes [25, 32]. Accordingly, we refrain from attributing the moderation effect to “protective” policies per se and instead interpret it as evidence that living in a more affirming *policy climate* dampens the translation of stress into psychological distress.

Third, and most notably, we identified meaningful subgroup variation in these effects: the protective impact of inclusive policies was particularly strong among divorced SGM individuals compared to their married counterparts. This novel finding suggests that inclusive policy environments may be especially important for SGM individuals who lack the social or legal protections that marriage can confer. Future research should explore the mechanisms underlying this differential effect, including the potential roles of social isolation, economic vulnerability, or legal precarity among divorced SGM populations. Although the intraclass correlation coefficients (ICCs) for depression and anxiety were small (< 1%), this pattern is common for psychological outcomes and does not invalidate cross-level inference. Low ICCs indicate limited between-state variance in average symptom levels but do not preclude meaningful heterogeneity in slopes: the target of our cross-level interaction [17]. Multilevel research increasingly emphasizes that substantial moderation can occur even when intercept variance is minimal, because structural features may shape how strongly individual stress translates into symptoms rather than baseline symptom levels themselves [18]. Therefore, the significant policy–stress interaction in our models is consistent with contemporary multilevel theory.

The finding that the buffering effect of inclusive policies was particularly pronounced among divorced SGM individuals may reflect how policy protections interact with social position to shape the intensity of minority stress. This dynamic is consistent with Brooks’ [7] emphasis on structural access and Diamond & Alley’s [12] framing of policies as mechanisms that create or dismantle social safety. In summary, our results highlight the multilevel nature of minority stress and underscore the critical role of structural interventions in reducing mental health disparities.

Neurobiological research offers further context for these findings. For example, Hatzenbuehler et al. [10] found reduced cortisol reactivity among SGM individuals living in states with more inclusive policies. Although we did not examine biological mechanisms directly, the psychological buffering effects observed in our study may reflect similar stress-regulatory pathways, potentially translating to clinically meaningful reductions in depression and anxiety at the population level.

While this study did not detect statistically significant three-way interactions by gender identity or sexual orientation, the observed main moderation effects are likely to be particularly important for individuals with multiple marginalized identities. Transgender, non-binary, bisexual, asexual, and queer individuals face unique and layered forms of minority stress, including healthcare discrimination, identity erasure, and limited social support [1, 3]. Although our models lacked sufficient power to fully explore these subgroup dynamics, prior research underscores that inclusive policies can reduce baseline disparities in depression and anxiety, especially in protective policy environments [33]. Thus, the overall buffering effect of policy inclusivity observed in our study may represent an even more critical protective factor for SGM subgroups at heightened risk for mental health disparities.

Transgender and non-binary individuals face distinct barriers to mental health, including discrimination in healthcare, employment, and social services. Consistent with prior research [1], our regression model indicated substantially higher depressive symptoms among gender-diverse groups compared to cisgender men (the reference group). Specifically, non-binary individuals (β = 0.589, *p* < 0.001) and transgender individuals (β = 0.422, *p* < 0.001) had significantly higher coefficients for depression (Supplemental Table S2). However, our analysis did not detect statistically significant three-way interactions between policy, stress, and gender identity, which may be due to limited statistical power in subgroup analyses. While we cannot conclude that the buffering effect of inclusive policies was differentially stronger for gender-diverse individuals, the overall protective effect observed in the full sample may still hold important implications for these groups, given their elevated baseline risk. This interpretation is supported by prior research showing that access to gender-affirming care in policy-protective environments is associated with reduced depression disparities [33].

While supplemental analyses indicated disparities in baseline symptoms across demographic groups such as age and sexual orientation (see Table S2), these variables did not significantly moderate the relationship between policy, stress, and mental health in our primary models. This suggests that the protective effect of inclusive state policies may operate broadly across SGM subgroups, rather than being specific to certain demographic profiles. Future research with larger samples should investigate whether more subtle differential effects exist [3].

The strong association between inclusive policies and improved mental health outcomes highlights the urgent need for comprehensive legal protection. Research by the American Psychological Association [2] emphasizes that legal recognition and non-discrimination policies are key determinants of well-being for SGM individuals. By ensuring equal rights and fostering a culture of acceptance, policymakers can significantly reduce the mental health burden experienced by this population.

Our study offers important contributions to understanding how structural factors affect mental health among SGM individuals, but several limitations should be considered. First, self-reported measures may be subject to recall bias and social desirability effects. However, the PHQ-9/2 and GAD-7/2 instruments used here are well-validated and widely adopted in population health research. Second, the exclusion of U.S. territories may limit the generalizability of our findings to those regions. Third, this analysis does not account for possible effects that the COVID-19 pandemic and related measures (i.e., lockdown and social isolation) may have on individuals. Finally, the cross-sectional nature of the data precludes strong causal inference. Future longitudinal studies tracking policy changes, such as recent bans on gender-affirming care, could help clarify the causal pathways linking policy environments, stress exposure, and mental health outcomes. Several limitations warrant consideration. First, All of Us is a volunteer cohort and not nationally representative; our SGM sample overrepresents White and highly educated individuals. Second, the MAP scores reflect policy environments in 2020, but participants provided mental health data through 2022 during a period of rapid policy change, introducing potential temporal mismatch. Third, analyses were not preregistered, introducing analytic flexibility. Fourth, proximal minority stress processes were not measured. Fifth, the cross-sectional design precludes causal inference and cannot rule out selective migration. Finally, future work should incorporate negative control analyses comparing SGM and non-SGM groups to strengthen arguments for structural stigma effects. In particular, our SGM sample was disproportionately White, insured, and highly educated relative to national SGM demographics [34]. These sociodemographic differences may attenuate or obscure the experiences of SGM individuals facing greater socioeconomic marginalization, for whom policy environments may exert even stronger psychological impacts [11].

Longitudinal studies are essential to understanding how shifts in state policies influence mental health trajectories over time. Additionally, qualitative research exploring SGM individuals’ lived experiences in different policy environments can provide deeper insights into the mechanisms through which policies shape mental health outcomes. Expanding research in this area will contribute to more effective policy interventions aimed at reducing mental health disparities among SGM individuals.

By integrating these perspectives, this analysis bridges the gap between individual experiences and broader policy implications, offering a comprehensive understanding of the factors influencing mental health disparities among SGM individuals.

### Public health implications

Findings indicate that inclusive LGBTQ-related state policies are associated with lower levels of depression and anxiety among sexual and gender minority adults, and that such policies buffer the adverse effects of stress on mental health. Strengthening protective policy environments, alongside individual-level interventions, may help reduce mental health disparities. Public health strategies that integrate policy advocacy with community-based prevention and mental health services are needed to promote resilience and equity among sexual and gender minority populations.

## Supplementary Information

Supplementary Material 1.

## Data Availability

The data that support the findings of this study can be accessed via the All of Us Researcher Workbench (https://workbench.researchallofus.org/). The data are not publicly available and cannot be made available by the corresponding author on reasonable request due to privacy or ethical restrictions. At the time of publication, access to the All of Us Researcher Workbench is restricted to researchers whose institution has signed a data use agreement with All of Us (https://www.researchallofus.org/register/).
