# Supplementary material for: A multilevel analysis of LGBT-relevant laws and sexual and gender minority mental health: evidence for the protective role of inclusive policies
Source: BMC Public Health. 2026 Jan 30;26:733. doi: 10.1186/s12889-026-26309-4 (PMC12930852; doi:10.1186/s12889-026-26309-4)
Supplement: Supplementary file 1 — Supplementary Material 1. [file 12889_2026_26309_MOESM1_ESM.docx]

**Supplemental Table S1**

*Data source and measure specifics.*

| Covariates | Data source and measure specifics |
| --- | --- |
| **Individual level covariates** |  |
| Age | *Data source: “date of birth” from the AoU prepackaged concept set “Demographics”*  *Age was calculated by subtracting the birth year from the year of outcome measurement* |
| No more than 40 years |  |
| 40 – 65 years |  |
| Over 65 years |  |
| Gender identity | *Data source: AoU Survey “The Basics”* |
| Man | Participants who identified as Man only |
| Woman | Participants who identified as Woman only |
| Non-binary | Participants who identified as one or more of the following:  Non-binary, Genderfluid, Genderqueer, Gender variant |
| Transgender | Participants who identified as one or more of the following:  Transgender, Transgender man, Transgender woman, FTM / MTF |
| Other | Participants who identified as one or more of the following:  Two-spirit, Man and woman, Questioning or unsure of their GI,  None of the above and specified another GI, None of the above but did not specify their GI |
| Sexual orientation | *Data source: AoU Survey “The Basics”* |
| Heterosexual | Participants who identified as Straight only |
| Gay/Lesbian | Participants who identified as Gay and/or lesbian |
| Bi-sexual | Participants who identified as one or more of the following:  Bi-sexual  Polysexual, omnisexual, sapiosexual or pansexual  Mostly straight but sometimes attracted to people of their own sex  Do not use labels |
| Asexual | Participants who identified as one or more of the following:  Asexual  Do not think of themselves as having sexuality |
| Queer | Participants who identified as Queer |
| Other | Participants who identified as one or more of the following:  Two-spirit  Have not figured out or in the process of figuring out their SO  Do not know the answer  None of the above and specified something else  None of the above but did not specify their SO |
| Multi-SO | Participants who belonged to more than one of the above main SO categories |
| Race/ethnicity | *Data source: AoU Survey “The Basics”* |
| Non-Hispanic White | Participants who were non-Hispanic and white only |
| Non-Hispanic Black | Participants who were non-Hispanic and black only |
| Non-Hispanic Asian&NHPI | Participants who were non-Hispanic and Asian and/or Native Hawaiian and Pacific Islander (NHPI) |
| Non-Hispanic another race | Participants who were non-Hispanic and Middle Eastern or North African (MENA) or selected none of these fully describe me |
| Hispanic | Participants who were Hispanic only |
| Multi race/ethnicity | Participants who had more than one race/ethnicity |
| Nativity | *Data source: AoU Survey “The Basics”* |
| U.S. born |  |
| Foreign born |  |
| Education | *Data source: AoU Survey “The Basics”* |
| Less than a college degree | Participants whose education level ranged from “Never attended school” to “1-3 years after high school” |
| College graduate | Participants whose education level was “College 4 years or more (College graduate)” |
| Advanced degree | Participants whose education level was “Advanced degree (Master’s, Doctorate, etc.)” |
| Annual household income | *Data source: AoU Survey “The Basics”* |
| Less than $35,000 | Participants whose annual household income ranged from “Less than $10,000” to “$25,000 - $34,999” |
| $35,000 – $74,999 | Participants whose annual household income was “$35,000 - $49,999” and “$50,000 - $74,999” |
| $75,000 – $149,999 | Participants whose annual household income was “$75,000 - $99,999” and “$100,000 - $149,999” |
| $150,000 and more | Participants whose annual household income was “$150,000 - $199,999” and “$200,000 or more” |
| Missing/prefer not to answer | Participants who selected “Prefer not to answer” or skipped this question |
| Health insurance | *Data source: AoU Survey “The Basics”* |
| Private | Participants whose responses contained self-purchased insurance and/or insurance from their employer |
| Public | Participants whose responses contained Medicaid, Medicare, military health care, Veteran Affairs (VA) health care, and/or Indian Health Service. |
| Both | Participants whose responses included healthcare plans from both Private and Public categories |
| Other/none | Participants who selected “Any other type of health insurance” or “I don’t have health insurance” |
| Missing/prefer not to answer | Participants who selected “Prefer not to answer” or skipped this question |
| Marital status | *Data source: AoU Survey “The Basics”* |
| Married |  |
| Live with partner |  |
| Separated |  |
| Divorced |  |
| Widowed |  |
| Never married |  |
| State | *Data source: first three digits of zip code from the prepackaged concept set AoU “Zip Code Socioeconomic Status Data”* |
|  |  |
| **State-level covariates** |  |
| Mental health facility ratio | *Data source: Substance Abuse and Mental Health Services Administration (SAMHSA) National Mental Health Services Survey (N-MHSS) and National Substance Use and Mental Health Services Survey (N-SUMHSS (30-33)*  The total number of eligible mental health facilities was extracted for each state  The ratio was calculated in the number of facilities per 1 million people |
| Total population | *Data source:* *U.S. Census Bureau(34)*  Use the 5-year estimate |
| Median age | *Data source:* *U.S. Census Bureau(34)*  Use the 5-year estimate |
| Sex ratio | *Data source:* *U.S. Census Bureau(34)*  Use the 5-year estimate  Measured in males per 100 females |
| Partisan control | *Data source: National Conference of State Legislatures (NCSL) website(35)*  Note that Nebraska was unicameral, its state control was replaced by its governorship party |
| Democratic | Democracy held both legislative chambers and the governorship |
| Republican | Republic held both legislative chambers and the governorship |
| Divided | Governorship and one or both legislative chambers held by different parties |

**Supplemental Table S2**

*The effect of each covariate on depression and anxiety among All of Us participants from 2020 to 2022 at individual level.*

| Individual level covariates | Depression ($N$ = 6,430) | Anxiety ($N$ = 6,392) |
| --- | --- | --- |
| Stress | .076*** (.001) | .080*** (.001) |
| Age |  |  |
| No more than 40 years | ref | ref |
| 40 – 65 years | -.316*** (.026) | -.419*** (.026) |
| Over 65 years | -.668*** (.033) | -.829*** (.033) |
| Gender Identity |  |  |
| Cis-gender man | ref | ref |
| Cis-gender woman | .213*** (.026) | .335*** (.026) |
| Non-binary | .589*** (.052) | .641*** (.052) |
| Transgender | .422*** (.063) | .448*** (.064) |
| Other | .459** (.149) | .401** (.148) |
| Sexual Orientation |  |  |
| Heterosexual | ref | ref |
| Homosexual | -.112 (.095) | -.154 (.096) |
| Bi-sexual | .189* (.095) | .178 (.096) |
| Asexual | .280* (.118) | .162 (.119) |
| Queer | .405*** (.114) | .376** (.115) |
| Other | .164 (.113) | .128 (.114) |
| Multi | .042 (.197) | -.024 (.200) |
| Race/ethnicity |  |  |
| Non-Hispanic White | ref | ref |
| Non-Hispanic Black | -.054 (.057) | -.111 (.058) |
| Non-Hispanic Asian&NHPI | -.074 (.067) | -.017 (.068) |
| Non-Hispanic another race | .136 (.105) | .109 (.106) |
| Hispanic | .035 (.044) | .073 (.045) |
| Multi race/ethnicity | .189** (.073) | .098 (.073) |
| Nativity |  |  |
| U.S. born | ref | ref |
| Foreign born | -.189*** (.044) | -.148*** (.045) |
| Education |  |  |
| Less than a college degree | ref | ref |
| College graduate | -.236*** (.030) | -.166*** (.031) |
| Advanced degree | -.398*** (.030) | -.347*** (.030) |
| Income |  |  |
| Less than $35,000 | ref | ref |
| $35,000 – $74,999 | -.254*** (.033) | -.190*** (.033) |
| $75,000 – $149,999 | -.443*** (.033) | -.368*** (.033) |
| $150,000 and more | -.530*** (.038) | -.433*** (.039) |
| Missing/prefer not to answer | -.196** (.060) | -.159** (.061) |
| Health Insurance |  |  |
| Private | ref | ref |
| Public | .131*** (.031) | -.005 (.031) |
| Both | -.176*** (.046) | -.326*** (.046) |
| Other/none | .095 (.077) | .005 (.078) |
| Missing/prefer not to answer | -.085* (.003) | -.138*** (.033) |
| Marital Status |  |  |
| Married | ref | ref |
| Live with partner | .168*** (.037) | .155*** (.038) |
| Separated | .279** (.097) | .156 (.098) |
| Divorced | .286*** (.045) | .077 (.045) |
| Widowed | .078 (.084) | -.144 (.086) |
| Never married | .290*** (.029) | .160*** (.029) |

Note. The statistics are $\beta$ (SE). * $p$ < .05, ** $p$ < .01, *** $p$ < .001

**Supplemental Table S3**

*Participants count by state for depression and anxiety among All of Us participants from 2020 to 2022.*

| State | Depression ($N$ = 6,430) | Anxiety ($N$ = 6,392) |
| --- | --- | --- |
| Alabama | 210 | 211 |
| Alaska | 4 | 4 |
| Arizona | 357 | 360 |
| Arkansas | 9 | 9 |
| California | 1113 | 1089 |
| Colorado | 47 | 47 |
| Connecticut | 43 | 43 |
| Delaware | 5 | 5 |
| District of Columbia | 45 | 46 |
| Florida | 203 | 203 |
| Georgia | 190 | 188 |
| Hawaii | 4 | 4 |
| Idaho | 9 | 9 |
| Illinois | 427 | 427 |
| Indiana | 25 | 24 |
| Iowa | 9 | 9 |
| Kansas | 18 | 18 |
| Kentucky | 17 | 17 |
| Louisiana | 47 | 47 |
| Maine | 21 | 21 |
| Maryland | 87 | 87 |
| Massachusetts | 694 | 693 |
| Michigan | 334 | 321 |
| Minnesota | 105 | 105 |
| Mississippi | 23 | 23 |
| Missouri | 24 | 24 |
| Montana | 9 | 9 |
| Nebraska | 9 | 9 |
| Nevada | 17 | 17 |
| New Hampshire | 24 | 24 |
| New Jersey | 41 | 41 |
| New Mexico | 23 | 23 |
| New York | 436 | 437 |
| North Carolina | 41 | 42 |
| North Dakota | 7 | 7 |
| Ohio | 53 | 54 |
| Oklahoma | 12 | 12 |
| Oregon | 63 | 63 |
| Pennsylvania | 660 | 657 |
| Rhode Island | 17 | 17 |
| South Carolina | 15 | 15 |
| South Dakota | 6 | 6 |
| Tennessee | 60 | 60 |
| Texas | 160 | 160 |
| Utah | 18 | 17 |
| Vermont | 6 | 6 |
| Virginia | 67 | 67 |
| Washington | 90 | 89 |
| West Virginia | 5 | 5 |
| Wisconsin | 518 | 518 |
| Wyoming | 3 | 3 |
